# Iodine nanoparticles enhance radiotherapy of intracerebral human glioma in mice and increase efficacy of chemotherapy

**DOI:** 10.1038/s41598-019-41174-5

**Published:** 2019-03-14

**Authors:** James F. Hainfeld, Sharif M. Ridwan, Yaroslav Stanishevskiy, Rahul Panchal, Daniel N. Slatkin, Henry M. Smilowitz

**Affiliations:** 1 Nanoprobes, Inc, 95 Horseblock Rd., Unit 1, Yaphank, NY 11980 USA; 2 University of Connecticut Health Center, Department of Cell Biology, 263 Farmington Ave., Farmington, CT USA

## Abstract

Gliomas and other brain tumors have evaded durable therapies, ultimately causing about 20% of all cancer deaths. Tumors are widespread in the brain at time of diagnosis, limiting surgery and radiotherapy effectiveness. Drugs are also poorly effective. Radiotherapy (RT) is limited by dose to normal tissue. However, high-atomic-number elements absorb X-rays and deposit the absorbed dose locally, even doubling (or more) the local dose. Previously we showed that gold nanoparticles (AuNPs) with RT could eradicate some brain tumors in mice and many other preclinical studies confirmed AuNPs as outstanding radioenhancers. However, impediments to clinical translation of AuNPs have been poor clearance, skin discoloration, and cost. We therefore developed iodine nanoparticles (INPs) that are almost colorless, non-toxic, lower cost, and have reasonable clearance, thus overcoming major drawbacks of AuNPs. Here we report the use of iodine nanoparticle radiotherapy (INRT) in treating advanced human gliomas (U87) grown orthotopically in nude mice resulting in a more than a doubling of median life extension compared to RT alone. Significantly, INRT also enhanced the efficacy of chemotherapy when it was combined with the chemotherapeutic agent Doxil, resulting in some longer-term survivors. While ongoing optimization studies should further improve INRT, clinical translation appears promising.

## Introduction

For many years physicists knew that the radiation dose was amplified around high atomic number targets^1^. Cells grown on a gold foil and irradiated were killed 160 times more than without the gold^2^. If tumors could be loaded with an X-ray absorbing material, it could be used to locally increase the dose, making radiotherapy (RT) much more specific and effective. Gold nanoparticles (AuNPs) showed early promise in test animals in 2004^3^, and even achieved 50% eradication of advanced gliomas in a mouse model^4^. Extensive world-wide efforts to study and use high atomic number nanoparticle radioenhancement both *in vitro*, *in vivo*, and in silico have followed^5^. After many years, however, clinical translation of this technology has lagged due to some significant drawbacks of AuNPs. AuNPs that have a long blood half-life, needed for maximal intravenous tumor loading, typically result in liver, spleen and other tissue accumulation and poor whole body clearance. One study reported only a 9% decrease in AuNPs from the liver over a 6 month period^6^. Metal nanoparticles are also typically highly colored and at the high levels needed for therapeutic efficacy can permanently color the skin purple (chrysiasis)^7,8^. Similarly, persons ingesting silver nanoparticle supplements at high levels permanently turn blue^9^. Gold is also costly.

An alternative to gold is iodine. It absorbs X-rays well and compounds are virtually colorless and of relatively low cost. There may also be more degradative or clearance pathways for organic iodine than inert solid gold particles. Low molecular weight iodine contrast media are in heavy clinical use for heart and other vascular imaging^10^. These iodine agents were shown after intratumoral injection and radiation to suppress the growth of subcutaneous tumors in rabbits^11^. A phase I clinical trial of patients with brain tumors receiving a standard intravenous (IV) iodine contrast agent prior to RT showed safety, but efficacy was not apparent^12^. Standard iodine contrast media infused intravascularly into rats bearing gliomas during synchrotron RT showed benefit^13^. A phase I/II clinical trial for brain tumor patients has been underway since 2012 using 80 keV synchrotron stereotactic radiation therapy applied shortly after IV injection of standard iodine contrast media^14^. Tumor concentrations reached 0.2% iodine, and the procedure was deemed safe. However, the existing iodine contrast agents have a short blood half-life of about 45 seconds^15^. If irradiation is performed very soon after application there is not enough time for the iodine agent to optimally accumulate in tumors or adequately clear from normal tissues thereby limiting the effectiveness of this approach.

Here we report the use of a new iodine nanoparticle (INP) that is better suited to enhance tumor RT: It does not appear to be toxic, does not color the skin, has reasonable clearance from liver and spleen, and accumulates in gliomas^8^. We show here that Iodine Nanoparticle Radiotherapy (INRT) significantly extends survival in mice with orthotopic gliomas. Although drugs have notoriously failed to significantly benefit glioma treatment, we show here that INRT synergizes with the chemotherapeutic drug Doxil to produce longer-term survivors. The use of INPs appears to overcome many of the problems with AuNPs, making clinical translation more feasible.

## Results

Basic information about our novel INP has been reported recently^8^. Briefly it is a crosslinked triiodobenzene molecule with a PEG coating, having a hydrodynamic diameter of about 20 nm, a blood half-life of 40 hours (1.7 days), and shows no obvious toxicity after an IV dose of 4 g iodine/kg body weight. INP treated mice showed the same weight gain as age-matched controls, complete blood count (CBC) and blood chemistry tests were normal, and histopathology of major organs showed no signs of inflammation or fibrosis.

**Figure 1:**
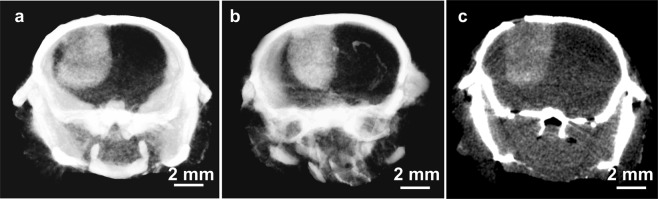
MicroCT images of human U87 gliomas in nude mice contrasted after intravenously injected INPs (3.5 g iodine/kg). (**a**) 24 hours after injection and (**b**) (a different mouse) 3 days after injection. (**c**) Single 65 µm section through center of tumor shown in (**a**).

Imaging by microCT indicated specific tumor localization in orthotopic human U87 gliomas growing in nude mice (Fig. 1). By calibrating the microCT with standards, delivery to tumors was quantified. After a well-tolerated IV injection of 3.5 g iodine/kg body weight, gliomas accumulated 0.55 ± 0.08% iodine (by weight) after 24 hours and 0.60 ± 0.03% (n = 3) after 3 days. This loading of iodine is calculated to give a radiation enhancement of about 2-fold^16,17^. Figure 1c is a 65 µm microCT section through center of the tumor shown in Fig. 1a illustrating that the iodine infiltrates even the center of the glioma.

**Figure 2:**
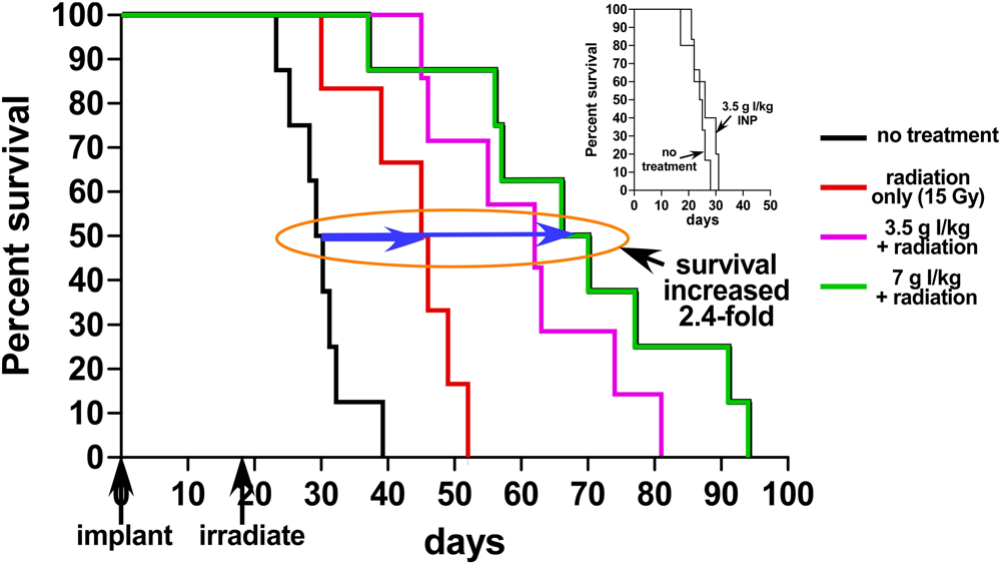
Kaplan-Meier survival graph showing survival vs. days after implantation of orthotopic U87 tumors. Radiation only (15 Gy, n = 6) extended life on average (at the 50% level, blue arrow) by 16 days (compared to no treatment, n = 8), but 7 g I/kg INP + 15 Gy (n = 8) extended life by 38 days (2.4-fold). Inset shows the INPs without radiation had no significant effect on survival. The INP results are statistically significant compared to RT only: P = 0.010 for RT vs. 3.5 g I/kg + RT and P = 0.0018 for RT vs. 7 g I/kg + RT.

The radioenhancement effect is proportional to the concentration of high atomic number enhancer present. Since a dose of 7 g I/kg had not shown any signs of toxicity, both 3.5 and 7.0 g I/kg were used and delivered by IV tail injection. Twenty four hours later, mice were irradiated with 15 Gray (Gy) from a 100 kVp X-ray source. The survival results are shown in Fig. 2. Results showed that radiation only (15 Gy) extended life on average by 16 days, but 7 g I/kg with RT extended life by 38 days, 2.4-fold compared to RT only (blue arrows, Fig. 2). INPs by themselves without RT had no effect on increasing or decreasing survival (Fig. 2 inset).

**Figure 3:**
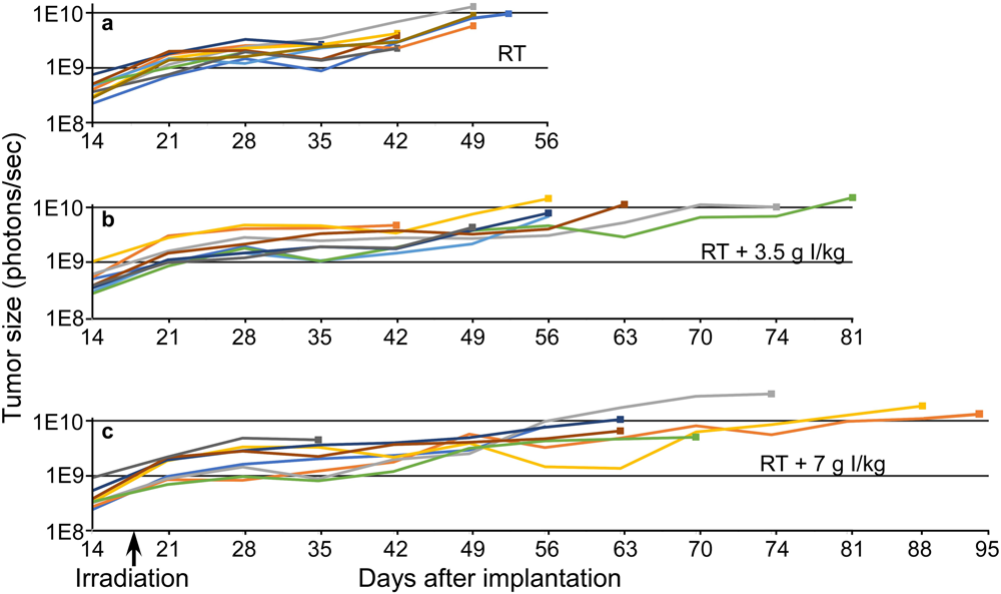
IVIS tumor size measurement for the 3 experimental groups. Each line represents an individual mouse. RT was 15 Gy given on day 18.

This trial was also followed using the *in vivo* imaging system (IVIS). U87 tumor cells had been transduced to express firefly luciferase that produced luminescent light in the presence of luciferin where light intensity is proportional to the number of viable tumor cells^18,19^ (Fig. 3). What is striking about these data is that the tumor cell count did not substantially shrink. This is surprising since one might expect the radiation, and more so the radiation plus iodine to be more effective at killing a larger fraction of tumor cells. There appears to be continued growth immediately after the irradiation for about 1–1.5 weeks, then negligible growth over the next 1–1.5 weeks, followed by a resumed progression of tumor growth. In this last phase, the iodine treated tumors appear to grow more slowly, thus extending the survival time. Up until about 3 weeks (day 37) from the time of irradiation it is difficult to see substantial differences in tumor progression between the 3 groups. The unirradiated animals (not shown) had unabated tumor growth and died soonest.

**Figure 4:**
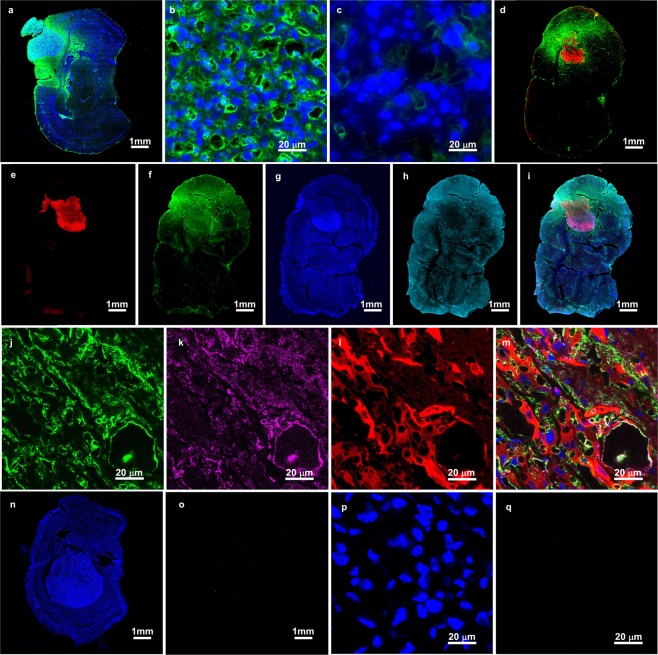
Fluorescently labeled coronal histological sections of mouse brains bearing U87 tumors. INPs were IV injected 24 hours prior to perfusion-fixation. (**a–c**) Sections from one mouse stained with an anti-PEG antibody (green) to show the INPs and DAPI (blue) staining cell nuclei. a: whole brain section showing INPs are localized to the tumor mass and edematous surrounding. (**b**) Higher magnification in tumor region showing the INPs (green) are largely staining blood vessels and capillaries. (**c**) Same magnification as (**b**), but in non-tumor region showing virtually no INP uptake. (**d**) In a second mouse whole brain section using U87 tumors transduced with Tomato red fluorescent protein (red) and stained with anti-PEG (green) for INPs. (**e**–**i**) Section from brain tumor in a third mouse using four colors: (**e**) tumor cells (red), (**f**) INPs stained with anti-PEG (green), (**g**) cell nuclei stained with DAPI (blue), (**h**) endothelial cells stained with anti-CD31 (light blue); (**i**) merged image. Again, INPs are localized to the tumor region (**e**,**f**). (**j**–**m**) Higher magnification in tumor region of third mouse. The INPs (green, **j**) are largely co-localized with tumor endothelium (violet, **k**). Tumor cells are shown in (**l**) (red). (**m**) Is a merged image showing INPs (green) around blood vessels and capillaries and in proximity to tumor cells (red), but largely not internalized in tumor cells. Controls without INP had no PEG staining (**n**–**q**): (**n**) whole brain stained with DAPI and anti-PEG, (**o**) same section as (**p**) with anti-PEG (green) channel only, (**p**) higher magnification of tumor region from section shown in (**n**) stained with DAPI and anti-PEG, (**q**): same section as (**p**) with anti-PEG (green) channel only.

A possible explanation of this behavior may be found from our histological study. An antibody to PEG was used to localize the pegylated INPs (Fig. 4). The INP staining (green) is confined to the tumor region but significantly also infiltrates the edematous peritumoral margin of the tumor where spreading glioma cells are known to be (Fig. 4a,d,f)^20–22^. At higher magnification, the INPs appear to mainly stain the endothelial cells (Fig. 4b), with less but some staining directly around tumor cells, and little staining inside tumor cells.

**Figure 5:**
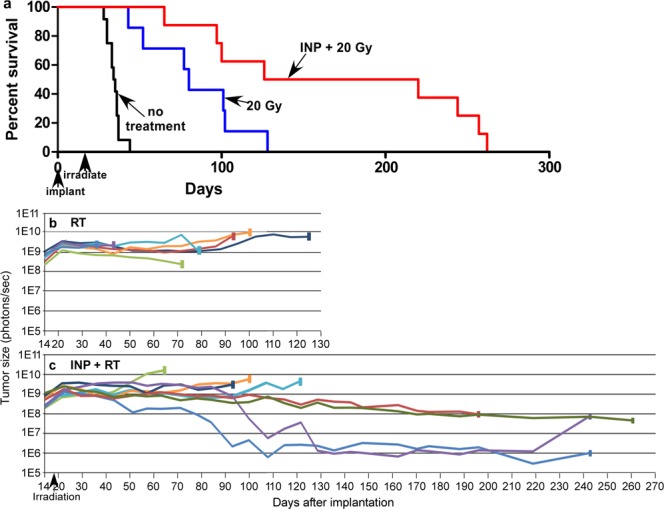
(**a**) Survival graph showing no treatment (n = 12), 20 Gy (n = 7), and INP + 20 Gy (n = 8) groups. (**b**,**c**) IVIS tracking of tumor cell viability for (**b**) 20 Gy, (**c**) 7 g I/kg INP + 20 Gy. Individual mice plotted. Terminal rectangles indicate animal deaths.

An interesting effect was seen when the dose was raised to 20 Gy. At this level, radiation alone extends survival (Fig. 5), with tumors growing more slowly (than at 15 Gy), but eventually tumor overgrowth and death result. Once again, a significant decrement in tumor cell count did not occur after RT alone (Fig. 5b). However, in the RT + INP group some of the animals (25%, 2/8) had tumor counts that decreased by more than 1000-fold (Fig. 5c), producing more durable life extension.

**Figure 6:**
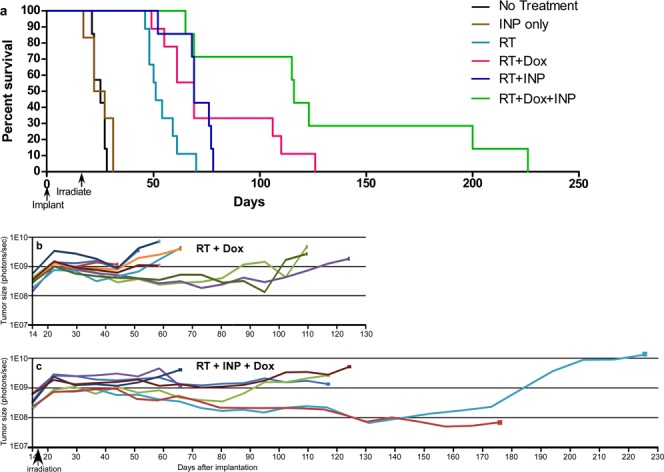
(**a**) Survival graph showing results with Doxil and INRT (15 Gy). Groups: no treatment (n = 7), INP only (n = 6), RT (n = 9), RT + INP (n = 7), RT + Dox (n = 9), RT + Dox + INP (n = 7). (**b**,**c**) IVIS signals proportional to viable tumor cells following RT + Dox (**b**) and RT + INP + Dox (**c**). RT was 15 Gy, INP was 7 g I/kg. Each line represents one mouse.

Since INRT locally increases tissue damage, we think largely to endothelial cells (Fig. 4b,j,k), one might expect INRT to enhance penetration and accessibility of drugs and cells of the immune system. Therefore it was logical to combine INRT with chemotherapy or immunotherapy to provide additive and perhaps synergistic benefit. To explore this hypothesis we tested INRT with a commonly used chemotherapeutic agent, Doxil. The rationale was based on the fact that drugs have poor accessibility to brain tumors, but INRT might disrupt more of the tumor endothelial barrier, enabling better drug penetration. Additionally, Doxil was chosen since it is a liposomal encapsulation of doxorubicin and has a longer blood half-life than free doxorubicin and is administered over several days, thus increasing the probability of matching the timing of the disrupted endothelial barrier kinetics with the drug availability in the blood. A RT dose of 15 Gy was used, and the resulting survival study is shown in Fig. 6a. At the 50% survival level, a value of 0.20 for the coefficient of drug interaction (CDI) is calculated^23^, indicating a strong synergy between the INP and Dox combination with RT. Once again, the IVIS signals are informative of the number of viable tumor cells (Fig. 6b,c). For most of the animals, there was no significant decrement in the number of tumor cells. However, in the RT + Dox and RT + INP + Dox groups some mice showed a 2–10x reduction in tumor load before progression. In the RT + INP + Dox group, tumor growth was slowest and 2/7 of the mice showed a more protracted ~10-fold reduction in tumor cells and concomitant longer survival. One of these mice later showed tumor regrowth, and both mice eventually died.

In all of the studies the whole mouse was imaged by IVIS and no sign of metastases to other parts of the body was found.

## Discussion

RT is used in about 50% of all cancer treatments^24^; improving it would benefit many patients. However, previously described physical radioenhancers have various drawbacks. Small molecule materials (such as standard iodine contrast media and gadolinium chelates) clear the body rapidly, do not optimally load and penetrate tumors, have rapid tumor washout, and additionally have high backgrounds in normal surrounding tissues at early times after injection. Nanoparticle agents with a long blood half-life can achieve higher tumor loading with lower backgrounds, but also largely accumulate in liver, spleen, and other tissues mainly being ingested by macrophages (the mononuclear phagocytic system, MPS). Many metal nanoparticles, including AuNPs, are highly colored and for the high tumor concentration needed, high levels must be administered for IV delivery, resulting in permanent skin discoloration, in addition to poor whole body clearance and high cost (e.g. with gold). The INPs described here appear to overcome many of the problems of other radioenhancers and have these desirable properties:, (1) non-toxic at very high levels (7 g I/kg), (2) colorless, (3) highly X-ray absorbing, (4) long blood half-life (40 hrs), (5) high uptake in tumors, (6) slow but consistent liver clearance (50% by six months, 70% by 12 months), (7) organic (not non-degradable metal) and (8) reasonable in cost^8^.

The brain and brain tumor INP distributions were remarkable. The INPs were specifically localized to the tumor region. A central tumor section (Fig. 1c) showed the iodine to be not completely uniform but essentially similar in the tumor interior and its periphery. This is quite different from the AuNP distribution seen in subcutaneous tumors where, after IV injection, the particles are mainly concentrated in the tumor periphery growing edge^25^. It is also quite different from the distribution found for the drug erlotinib (an EGFR inhibitor) that showed 4.7-fold higher concentration in the tumor core compared to its periphery^26^ in the same U87 tumor model. Such drugs were found to have uptake and localization strongly modulated by efflux transporters. Since this tumor periphery depletion was not seen with the INPs, it suggests that these drug efflux transporters may not affect the INP localization, therefore overcoming a drawback with drug delivery.

From the higher resolution histology (Fig. 4b) it appears that by 24 hours post IV injection the iodine is in highest concentration in the endothelial cells suggesting less access to the glioma cells themselves. Since the radiation enhancement effect is highly confined, the iodine must be close to the cell nucleus for the best effect^27^. The major effect may therefore be more killing of tumor-associated endothelial cells rather than the tumor cells themselves. This is also consistent with radiation studies that show angiogenic endothelial cells are damaged at >10 Gy whereas normal tissue endothelium is much less sensitive, thus making radiation more effective in tumors^28^.

Other approaches have been studied to overcome nanoparticle pharmacokinetic and distribution problems. For example direct intratumoral injections can obviate systemic toxicity, skin coloration, poor clearance, low tumor uptake, and cost issues. Although this may lead to clinically palliative results, the injected material takes the path of least resistance and leaves some tumor regions under treated, allowing recurrence^29,30^. Furthermore, direct intratumoral injection is not practical or ideal for most clinical cancers where the tumors are heterogenous in size, disseminated widely, range from microscopic to macroscopic and are typically deep in the body. IV administration provides better access to tumors, but comes with additional problems to overcome such as toxicity, clearance, and skin discoloration.

An interesting observation in this study was the general lack of tumor cell killing (Figs 3,5, and 6). This is somewhat surprising since the radiosensitivity of the U87 tumor line used has been reported, with only ~1/100 of the cells surviving after ~12 Gy *in vitro*^31^. For the experiment shown in Fig. 3 we used 15 Gy since a single 15 Gy dose is reported to be equivalent to the 60 Gy given in 30 fractions used clinically to treat gliomas^32^. However, the number of tumor cells did not decrement substantially after irradiation (Fig. 3). This is radically different from the *in vitro* result. Possible explanations could be 3-dimensional contact inhibition, slower growth rate *in vivo* (reducing radiosensitivity), tumor microenvironment, and the presence of free radical scavengers *in vivo*^33^. Even at 20 Gy there was no significant tumor cell killing from the radiation only (Fig. 5b). It is clear that these glioma cells become much more radiation resistant when grown intracranially. Nevertheless, tumor growth was significantly delayed with INRT (cf. Fig. 3a,c). One explanation of these results is that the endothelial cells are the prime radiation target. Tumor microvessels have been found to be considerably more radiosensitive than normal blood vessels^28^. A previous study reported that irradiation with 15 Gy in a single dose to human glioblastoma xenografts grown intracranially in nude mice (U87, same as this study) profoundly reduced endothelial cell numbers and decreased blood perfusion to 10% of control^34^. That study found recurrence was fueled by recruitment of bone marrow-derived cells (BMDCs) restoring blood flow (vaculogenesis and not angiogenesis) and this could be reduced by inhibition of HIF-1 and other substances that block BMDCs. However, even with such drugs, gliomas remain difficult to treat. It appears that even if vasculogenesis is inhibited and life gets extended, eventually tumor vasculature is slowly rebuilt and tumors resume their rapid growth rate, causing demise. Many preclinical therapy reports do not include IVIS data (that would report on actual tumor viability), but instead report short-term (~30 day) slowing or even cessation of tumor growth with some new drug or treatment. These results may similarly be due to a killing of the more sensitive tumor microvessels and not the tumor cells themselves. If the tumor cells are not eliminated, recurrence will probably eventually occur. A popular theory is that since up to 2,000 tumor cells are supplied by one endothelial cell, killing one endothelial cell will therefore kill many tumor cells^35^. To the contrary, our data presented here seem to render this approach largely invalid. Even though endothelial damage is substantial, it appears there is enough blood flow to keep nearly all the tumor cells alive, but in a dormant hypoxic and malnourished state. Also, the histology shown in Fig. 4b seems to indicate only a few tumor cells per endothelial cell (rather than thousands), which also means there is a significant overall and collateral blood supply. Figure 4b also shows the highest INP concentration to be in the endothelial cells rather than surrounding or inside tumor cells. Thus in all likelihood the INPs will enhance endothelial cell kill, thus requiring more time for repair and hence providing more life extension. A better answer to long-term survival would be to eradicate the tumor cells themselves rather than just the tumor endothelium. Surprisingly, this appears to have happened in some mice when we increased the radiation dose to 20 Gy (Fig. 5c); 25% of the animals showed a decrement of ~1,000-fold in tumor cells, whereas this was not seen in the RT only group. We suggest this is due to the iodine concentration and radiation dose combination reaching the threshold for tumor cell eradication. With such a reduction in viable tumor load, longer term survival results. Future work is focused on further enhancing this effect.

RT is also synergistic with some chemotherapy agents^36^. RT applied before drug administration has been shown to enhance drug delivery^37^. Presumably radiation disrupts the endothelial and perhaps other barriers, enabling better penetration and tumor access of the drugs. This effect is demonstrated by the results reported here where a single course of Doxil treatment was combined with INRT (Fig. 6). Importantly, significant tumor reduction (about 10-fold) was obtained in 29% (2/7) of the mice, although one of these mice later showed tumor regrowth and both eventually died. However, once again, significant tumor kill (as opposed to endothelial damage) results in more sustainable long term survival. INRT may also add significant benefit when combined with other drugs, perhaps reviving their usefulness despite their sub-optimal solo performances.

The studies here were done using a human glioma in a nude (athymic, immunodeficient) mouse. An active immune system might be expected to result in even better responses. RT has been shown to enhance the immune response^38^ and is known to be synergistic with checkpoint blockade^39,40^. Accordingly we predict INRT should further improve immunotherapy enabling greater penetration and interaction of the immune system with the tumor.

A controversial issue is: How effective is the Enhanced Permeability and Retention (EPR) effect in humans? Most preclinical studies are done in rodents where xenograft tumors generally grow more rapidly compared to tumors in humans. Rapidly growing tumors have more angiogenesis, more immature vessels with less basement membrane backing, and these are leakier to nanoparticles up to ~200 nm which are then retained. Since tumors are typically the only site of angiogenesis, EPR can be an important mechanism for specific nanoparticle tumor delivery. An extreme opinion proffers that EPR is completely absent in humans^41^. Although few human studies addressing this issue have been done, a radiolabeled liposome study in patients found specific tumor loading^42^. Moreover, the standard method of definitively diagnosing a brain tumor is a Gd-enhanced MRI scan, where the contrast agent leaks out where the tumor is (demonstrating EPR in humans). Brain tumors, however, do vary in their leakiness, with ependymomas having an almost intact blood brain barrier (BBB), but most other brain tumors have altered or disrupted BBB. The most prevalent and deadly type of brain tumor, glioblastoma multiforme (GBM), is highly edematous with notoriously leaky disrupted endothelium^43^. Migrating glioma cells travel along blood vessels, stripping off astrocyte end feet controlling the normal endothelial BBB. Studies have shown that “importantly, single glioma cells are sufficient to locally open the BBB”^44^, allowing penetration of appropriate agents, perhaps such as INPs^29^. Most recurrences come from migrating glioma cells^20^. The INRT approach could increase the radiation dose to them and might give hope to better treating these migrating and, so far, intractable-to-treat and deadly cells.

The strategy used here is to safely load the tumor with a high enough iodine concentration to significantly increase the radiation dose enhancement factor. This requires a rather large concentration of heavy atoms (~0.7%)^16^. If the material is delivered IV (better tumor coverage than by direct intratumoral injection^38^), a substantial amount is needed, since nanoparticles typically only accumulate in tumors 1–15% ID/(g tumor)^45^. Here we achieved with INPs 6.9% ID/g. This is a physical method quite different from drug therapy where only micro or milligrams of compounds are effective. Nevertheless, both approaches may be limited by toxicity at higher levels. Our previous work showed the INPs to be non-toxic at 4 g I/kg^8^, which might be compared to drugs being non-toxic at their respectively lower doses. In any case, lower doses of any therapeutic would be desirable. For INPs a next step will be targeting them. For example, RGD nanoparticle targeting was found in some cases to increase tumor uptake 3–5-fold^45^.

Active targeting to tumor markers using antibodies, peptide, aptamers, compounds, or drugs may improve delivery. However, a recent literature meta-analysis found that active targeting on average only increased tumor loading by 50% and clinical trials often showed little benefit compared to passive targeting^46^. This may be due to penetration barriers such as the endothelium, poor blood supply to parts of the tumor, or tumor microenvironment components such as collagen or hyaluronic acid that become the limiting factors to accessing tumor cell surface markers. Nevertheless, even when the tumor uptake is the same with or without active targeting, active targeting promotes internalization of nanoparticles into tumor cells^47^. For physical dose enhancement by iodine or metal nanoparticles, this can be a critically important factor since the dose enhancement factor increases sharply with proximity to the nucleus^48^. Other approaches to improve nanoparticle delivery include tumor vessel normalization to improve tumor perfusion, vascular promotion agents to increase leakiness, stroma-depleting agents, and reduction of tumor inflammation^49–52^.

Materials with elements that highly absorb X-rays are potent radioenhancers, but their effective clinical translation has been virtually non-existent. AuNPs have been extensively studied for over 14 years^3^ (recent review^5^) and other nanoparticles containing gadolinium^53^, bismuth^54^, titanium^55^, thulium^56^, platinum^57^, and hafnium^58^ have also been investigated. Iodine contrast media were used in clinical trials first by Norman and coworkers^12^, then later by Adam and coworkers^14^. Impediments to translation of these radioenhancers include: (1) poor tumor uptake, (2) toxicity at the high levels needed for IV application, (3) poor whole body clearance, (4) low applicability and effectiveness of direct intratumoral injections, (5) permanent skin discoloration for many metal nanoparticles, and (6) high cost. Here we have tried to overcome these obstacles by using INPs^8^. In the iodine clinical trial of Adam *et al*.^14^, standard contrast media reached a level of 0.2% in gliomas, and irradiation had to be done immediately after IV infusion due to the short blood life and short tumor retention of the iodine compound. Additional RT fractions would require additional injections. However, with the INPs studied here, tumor accumulation was about a factor of 3 higher (0.6%) and due to long blood life and tumor retention, it was nearly constant for at least 3 days (the longest measured) which would facilitate fractionation and remove the necessity of precisely timing the irradiation with the injections. However, uptake of the INPs in human gliomas remains to be tested for an accurate comparison. Nevertheless, the advance of developing a potent INP that appears to overcome the obstacles of previously tried physical radioenhancers may bring this technology closer to clinical translation.

For clinical translation, an issue to address is the X-ray energy. In the studies here and many other dose enhancement studies with gold and gadolinium nanoparticles, kilovolt (kV) X-rays have been used, whereas in the clinic, 6–25 million volt (MV) photons are almost exclusively used. Monte Carlo calculations predict that gold with ~100 kVp X-rays will increase the dose ~180–300 times more than with 6 MV photons^16,59^, since the absorption and photoelectric effect is strongest in the kV range. However, a number of cell and animal studies have shown significant dose enhancements with 6 MV with low concentrations of gold, some even showing only a 2-fold reduction in effect going from kV to MV^60–63^. The explanation invoked is that there are additional biological and physical responses that are not fully understood. Therefore the INPs may be effective with MV photons, but this remains to be tested.

X-rays only in the othovoltage kV range (<400 kV) were used for cancer therapy in the first part of the 20^th^ century, but had a disadvantage for deep tumors due to exponential dose drop-off with depth giving high entrance (skin) doses, a problem for treating deep tumors. About 1950, engineers were able to make million volt power supplies using linear accelerators (LINACs) and used them to generate high energy X-rays, first used on a patient in 1953. The interaction with tissue is less and builds up beneath the skin (with intensity peaking at ~1 cm, then ~exponentially decreasing), thus sparing the skin. Radiation oncologists discarded their kV machines for LINACS. However, there is now a resurgence in the use of kV radiation: Grid, minibeam and microbeams were discovered that showed tissue irradiated with small intervening unirradiated gaps fully recovers, thus avoiding entrance dose damage^64–66^. Even ultrahigh-dose millimeter- and micrometer-wide orthovoltage X-ray beams have surprising yielded minimal normal-tissue damage: intervening minimally irradiated tissues enable almost full repair of many kinds of normal tissues^67–69^. The beams can be interlaced or converged at the tumor, providing an even better dose distribution than with MV X-rays^70^. This technique is now being considered for improving synchrotron and proton radiotherapy^71–73^.

A second possibility for using kV X-rays with the INPs is to use stereotactic synchrotron irradiation (which is in the kV range) where a deep tumor is irradiated from multiple directions, thus spreading the incident (skin) dose over a larger area, but the dose is combined at the tumor focal point. A clinical trial for brain tumor patients is already underway using 80 keV synchrotron stereotactic radiation therapy applied shortly after intravenous injection of standard iodine contrast media^14,74^, showing that clinical translation with kV photons and iodine is now a reality.

A third practical way to use orthovoltage for clinical brain tumor irradiation is by using a standard CT scanner (even without modification). One group studied this and found it to be very feasible^17^. They calculated that 0.5% iodine would approximately double the tumor dose, and that normal brain tissue could be spared. Also, standard CTs deliver ~2 Gy/min, which would accommodate normal fractionation regimens. Furthermore, a clinical trial was already run using brain tumor patients, low molecular weight standard iodine contrast media (Iopamidol) with a CT for irradiation that demonstrated safety^12^.

**Figure 7:**
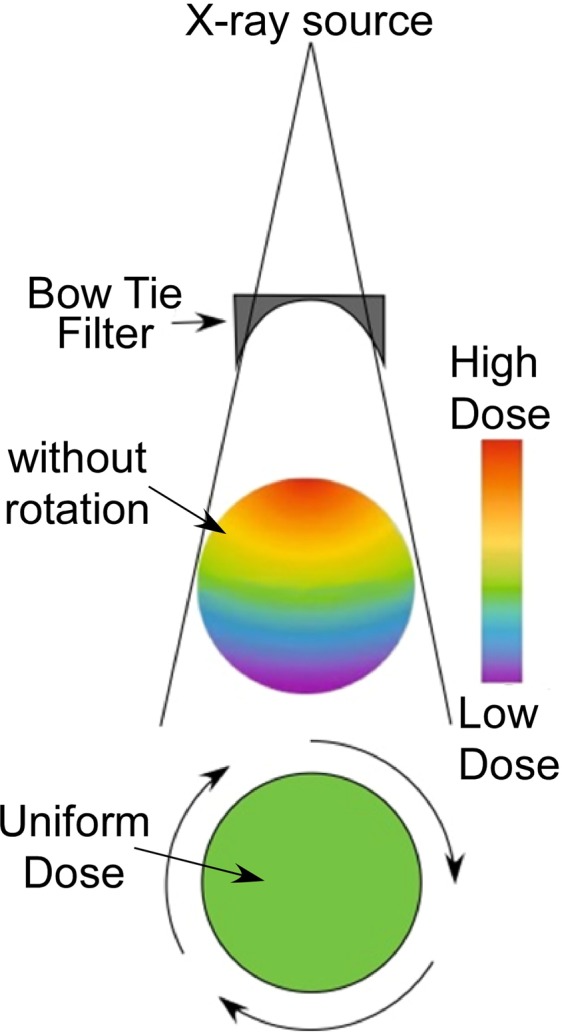
A standard CT with a standard bow-tie filter reduces intensity towards the edges such that upon rotation (i.e., normal scanning) a completely uniform dose is created throughout the subject, thus eliminating the dose-depth fall-off problem.

We also point out that the perceived dose-depth ‘problem’ is only for unidirectional irradiation. However, CT makers have had to deal with dose/absorption variations affecting image quality since X-rays passing through the center of the subject in a fan beam have greater absorption than at the edges. The solution to normalizing image signal to noise is a bow-tie filter and tomo-irradiation (rotation). This ideally produces isodose across the whole subject and is what is standard on all CT scanners without modification^75,76^ (Fig. 7). Hence, with a CT and standard bow-tie filter there is no dose-depth problem. This is highly relevant to the treatment of brain metastases where the standard of care is whole brain radiation. By increasing the dose only at tumor locations due to specific INP loading, a better outcome may be expected.

Therefore there are at least 3 ways to overcome the depth/dose issue with kV X-rays: (1) minibeams, (2) stereotactic irradiation, or (3) use of a CT. Although kV beams appear best, significant MV enhancement also appears possible.

## Conclusion

This is the first report of novel iodine nanoparticles (INPs) being used for glioma therapy in a mouse model. After intravenous injection, the non-toxic 20 nm nanoparticles targeted brain tumors. It was found that radiotherapy + iodine nanoparticles increased life by more than 2-fold compared to RT alone and also greatly improved drug potency when combined with Doxil. This new iodine nanoparticle appears promising for clinical translation and might significantly improve and complement the limited treatment options currently available for brain tumors and other cancers.

## Methods

### Institutional Animal Assurance

Animal experiments were conducted according to NIH guidelines and approved by the institutional animal care and use committee of University of Connecticut Health Center before start of the study. Protocol 101823-0521, Radiation therapy, imaging and adjunct prophylaxis, was last approved 8/27/2018. Six to eight week old female athymic nude mice (~20 g) were purchased from Envigo (6903FHSD Athymic Nude FOXNLNU) and Charles River NCI (NCI-553 Athymic Nude NCR Nu/Nu) and were generally used within a week or two of receipt for these experiments.

### Cell Lines

All of the therapy experiments were done with the U87 luciferase transductant purchased from Perkin Elmer. The cells were reported to be negative for mycoplasma contamination by IDEXX BioResearch on 6/21/18. The cells obtained from Perkin Elmer were passaged <5 times prior to use. The immunofluorescence experiments were done using U87 cells that had been purchased from ATCC and subsequently transduced to express tomato-luciferase by the DNA/RNA Delivery Core at Northwestern University. These cells were reported to be free of mycoplasma contamination by IDEXX BioResearch on 6/21/18 and were passaged <5 times prior to use.

### Iodine nanoparticles

These are polymerized triiodobenzene with a PEG coating. Their synthesis is detailed in a prior publication^8^. Briefly, in a typical preparation, 870 mg (1.06 mmol) Iohexol (Medchem Express, Monmouth Junction, NJ) was oxidized with sodium periodate (MilliporeSigma) for 30 minutes, followed by rotary evaporation to dryness. The product was resuspended in water and polymerized with 43 mg (0.49 mmol) of carbohydrazide (MilliporeSigma). To the crosslinked particles, 3.2 g (3.2 mmol) of 1 kDa aminoPEG (Creative Pegworks, Chapel Hill, NC) was added and left to react overnight. Sodium borohydride (60 mg, 1.6 mmol, MilliporeSigma) was added and allowed to react for 3 hours. The particles were transferred to a tangential flow filter device (Pall Minimate) with a 50 kDa filter and washed with 15 L water. The particles were then concentrated to 70–80 mg I/mL and exchanged into phosphate buffered saline using 50 kDa Amicon centrifugal concentrators (MilliporeSigma). Concentration above 80 mg I/mL gave solutions that were not easily loaded into syringes with 28 gauge needles due to viscosity. The final product was light yellow in color and the yield of iodine in the final particles was typically 35% from the starting iodine.

### MicroCT Imaging and Quantification

Various times after IV injection (INPs at 70–80 mg iodine/ml), mice were euthanized (0.15 mL of 100 mg/mL ketamine intraperitoneally (IP)) and immediately imaged by microCT (Scanco Medical AG µCT40, Bruttisellen, Switzerland), operated at 70 kVp. The source spot size was 5 µm (with 0.5 mm Al filtering), sampling with 15 × 15 × 15 µm voxels in a 30 mm-diameter field. 3 mm stacks of 200 sections at 2000 projections per revolution and an integration time of 300 ms/projection were collected, each stack requiring 20 minutes. Images were quantified and presented using Amira software (Mercury Computer Systems, Chelmsford, MA). Standards were prepared in tubes filled with a range of sodium iodide and Iohexol concentrations. Quantification was done by averaging the intensity over tissue volumes and reading the value from the standard curve adjusted for uninjected tissue values.

### IVIS

An *In-Vivo* Imaging System (IVIS spectrum®, Perkin Elmer) was used to quantify viable tumor cells. Imaging was done 13 minutes after a subcutaneous injection of luciferin (37.5 mg/kg). Photon counts were proportional to the number of luciferase transfected U87 tumor cells. A signal of ~8.0 × 10^8^ photons/sec corresponded to a tumor of 2.5–3 mm diameter, which was confirmed by dissecting the brains of U87-RedFluc tumor bearing mice.

### Histology

Twenty four hours after an IV injection of 1.75 gI/kg INPs, mice were cardiac perfused and fixed. Brains were removed, cryopreserved and cryosectioned. 4,6-Diamidino-2-phenylindole (DAPI) staining was used for nuclei. Primary antibodies used were rabbit anti-PEG (1:500; Abcam Cat # AB512572) and goat anti-CD31 (1:100; Abcam Cat # 19808). Secondary Antibodies used were donkey anti-rabbit Alexa Fluor 488 (1:400; Invitrogen A21206), and donkey anti-goat Alexa Fluor 647 (1:200; Life Technologies A21447). Imaging was done using a widefield fluorescence microscope (Zeiss Axio Observer Z1) and a confocal fluorescence microscope (Zeiss LSM 880).

### Tumor implantation

Fifty to one hundred and twenty-five thousand human U87-MG glioblastoma cells (transfected with luciferase) were implanted 2.5–3.0 mm deep into the striatum of nude mice through a 0.5 mm diameter burr hole made on the left coronal suture of the skull 2/3 of the way between the midline and the temporalis muscle insertion^77^.

### Treatment/Irradiation

Tumors were grown to an advanced stage, reaching 5 × 10^8^–10^9^ photon counts per sec assessed by IVIS. The group treated with INPs at 3.5 g I/kg were IV injected with a concentration of 70 mg I/ml INPs in 2 injections spaced 3 hours apart. The group treated with 7.0 g I/kg were IV injected 4 times, 2 each day spaced 3 hours apart. Twenty four hours after the last injection, mice were irradiated with 15 or 20 Gy 100 kVp X-rays. Mice were anesthetized with 140 mg/kg ketamine and 3 mg/kg xylazine in phosphate buffered saline given IP in about 0.06 mL. The head and body were protected by a 3.4 mm-thick lead shield with a notch that enabled irradiation 8.0 mm caudally from the posterior canthus of the left eyelid and dorsally from the dome of the palate to above the calvarium. Irradiations used a Philips RT100 X-ray generator (Amsterdam, The Netherlands) operating at 100 kVp with a 1.7 mm Al filter. Dose was calibrated using a Radcal ion chamber (Monrovia, CA). To prevent lethal brain edema, dexamethasone (5 mg/kg) was injected subcutaneously 18 and again 6 hours before irradiation and 6 and again 18 hours after irradiation^78^. Mice were euthanized when they either lost 20% of their weight at RT or presented any sign and symptoms of sickness or increased intracranial pressure (seizure, motor deficiencies etc.) and were indicated with terminal squares as death in the Figs 3,5, and 6.

### Doxil

The group of mice treated with IV Doxil after radiation therapy, received a total dose of 15 mg/kg which was administered in 6 divided doses (2.5 mg/kg each dose) over two weeks^79^.

### Experimental Groups

Six to 12 mice were used per group to enable differences in treatment to be determined with statistical significance (P < 0.05) as determined using the G-power statistical program^80^. Tumors were monitored by IVIS. The mice were randomized into groups of mice with similarly sized tumor distributions. Since the tumors varied in size, the median tumor size in each group was about 7–8 × 10^8^ at the time of irradiation. Mice with extremely large tumors and extremely small tumors were excluded. Overall, tumors in each group ranged from 10^8^ to 10^9^ photons/sec at the time of irradiation

### Statistical significance

P values between experimental groups were calculated using the Log-rank (Mantel-Cox) Test using Graphpad Prism software.

## Data Availability

Explicit materials, data, and associated protocols are available from the corresponding author upon reasonable request.
